# MicroRNAs within the Basal-like signature of Quadruple Negative Breast Cancer impact overall survival in African Americans

**DOI:** 10.1038/s41598-022-26000-9

**Published:** 2022-12-22

**Authors:** Anusha Angajala, Hughley Raymond, Aliyu Muhammad, Md Shakir Uddin Ahmed, Saadia Haleema, Monira Haque, Honghe Wang, Moray Campbell, Rachel Martini, Balasubramanian Karanam, Andrea G. Kahn, Deepa Bedi, Melissa Davis, Ming Tan, Windy Dean-Colomb, Clayton Yates

**Affiliations:** 1 Department of Biology and Center for Cancer Research, Tuskegee University, Tuskegee, AL 36088 USA; 2 Department of Pathology, University of South Alabama, Mobile, AL 36604 USA; 3 Pharmaceutics and Pharmaceutical Chemistry, College of Pharmacy, The Ohio State University, Columbus, OH 43210 USA; 4 Department of Surgery, Weill Cornell Medicine, New York, NY 10021 USA; 5 Department of Pathology, The University of Alabama at Birmingham, Birmingham, AL 35249-7331 USA; 6 Graduate Institute of Biomedical Sciences and Research Center for Cancer Biology, China Medical University, Taichung, 406040 Taiwan; 7 Department of Hematology/Oncology, Piedmont Hospital, Newnan, GA 30265 USA; 8 Department of Biochemistry, Faculty of Life Sciences, Ahmadu Bello University, Zaria, 810107 Kaduna State Nigeria; 9 Bangladesh Council of Scientific and Industrial Research (BCSIR), Dhaka, Bangladesh; 10 Department of Pathology, Johns Hopkins School of Medicine, Baltimore, MD 21218 USA; 11 Cancer Genetics and Epigenetics, Sidney Kimmel Comprehensive Cancer Center, Johns Hopkins University School of Medicine, The Bunting-Blaustein Cancer Research Building 1, 1650 Orleans Street - Room 1M44, Baltimore, MD 21287-0013 USA; 12 Department of Urology, Johns Hopkins University School of Medicine, Baltimore, MD 21218 USA

**Keywords:** Cancer, Cell biology, Computational biology and bioinformatics, Immunology, Molecular biology, Biomarkers, Diseases, Health care, Oncology

## Abstract

We previously found that QNBC tumors are more frequent in African Americans compared to TNBC tumors. To characterize this subtype further, we sought to determine the miRNA–mRNA profile in QNBC patients based on race. Both miRNA and mRNA expression data were analyzed from TCGA and validated using datasets from the METABRIC, TCGA proteomic, and survival analysis by KMPLOT. miRNA–mRNAs which include FOXA1 and MYC (mir-17/20a targets); GATA3 and CCNG2 (mir-135b targets); CDKN2A, CDK6, and B7-H3 (mir-29c targets); and RUNX3, KLF5, IL1-β, and CTNNB1 (mir-375 targets) were correlated with basal-like and immune subtypes in QNBC patients and associated with a worse survival. Thus, QNBC tumors have an altered gene signature implicated in racial disparity and poor survival.

## Introduction

Breast cancer remains a leading cause of death among women globally due to its lack of response to therapy, poor prognosis, and low survival rate. Although breast tumors are typically categorized by multiple subtypes based on gene expression, women of African Ancestry are frequently diagnosed with TNBC, which is a heterogeneous disease that is characterized by the absence of estrogen receptor (ER), progesterone receptor (PR), and human epidermal growth factor receptor 2 (HER2). We recently identified that a subset of TNBC patients that also lack Androgen Receptor (AR) and we named them Quadruple Negative breast cancer (QNBC) and associated them with basal-like (BL1 and BL2) subtype, an immunomodulatory (IM) subtype^1,2^. Furthermore, these QNBC basal-like tumors are commonly found in younger AA women and have worse outcomes compared to other breast cancer subtypes, including TNBC tumors^1^. Hence, there is a need for identifying the underlying mechanisms associated with the QNBC subtype.

miRNAs are short, non-coding RNAs (~ 18–24 nucleotides) that modulate gene expression post-transcriptionally^3^ at the mRNA level. Under certain conditions, miRNAs can be either oncogenes or tumor suppressors, regulating several rate-limiting events in carcinogenesis, including sustaining cellular proliferation, escaping growth suppressors, apoptotic resistance, angiogenesis, and promoting invasion and metastasis^4^. A given miRNA can target multiple mRNAs^5^; and these mRNA–miRNA interactions can result in either reduced mRNA stability or inhibition of translation into proteins. In this way, miRNAs regulate gene expression and have clinical relevance as biomarkers^6–8^. Novel experimental therapeutics that target miRNAs are now gaining attention in preclinical studies and clinical trials^9,10^.

Here, we report that levels of miRNAs mir-17, mir-20a, mir-135b, mir-584, and mir-532 are overexpressed and levels of miRNAs mir-29c, mir-10a, and mir-375 are decreased such that these changes correlate with a lower survival for AA patients regardless of subtype. Furthermore, these miRNAs appear to target mRNAs of FOXA1 and MYC (mir-17/20a targets), GATA3 and CCNG2 (mir-135b targets), CDKN2A, CDK6, and CD276 (mir-29c targets), RUNX3, KLF5, IL1-β, and CTNNB1 (mir-375 targets), all these molecules have been associated with driving both the basal-like and immune gene signatures in QNBC tumors. These findings highlight a regulator role of miRNA associated with race and within the QNBC subtype.

## Materials and methods

### RNA sequencing, proteomics data analysis, and determination of AR status

BRCA miRNA quantification files, metadata, and clinical files for 1206 samples were downloaded from the TCGA data portal (https://portal.gdc.cancer.gov/) in 2016. There were 103 solid normal tissue samples, seven metastatic tumor samples, and 1096 primary tumor samples. The miRNA files were merged into a single file. The DESEQ2 package in R was used for normalization and for assessing differential gene expression. On January 13, 2017, BRCA gene expression data in the form of fragments per kilobase of transcript per million mapped reads upper quartile (FPKM-UQ) units and metadata were downloaded from TCGA^11^. For primary tumor samples (n = 1102), gene expression was evaluated. As previously described, quantile was used as a threshold to determine AR status^1^. AR expression greater than quartile 1 or 25th percentile was considered AR-positive. AR expression lower than quartile 1 or 25th percentile was considered AR-negative. For 105 primary tumor samples and three solid normal tissue samples, TCGA breast cancer proteomics data and associated clinical information files were downloaded from the Clinical Proteomic Tumor Analysis Consortium (CPTAC). Unshared log-ratio units were used for the protein expression of the genes^11^.

### Microarray data set analysis

Two Gene Expression Omnibus (GEO) datasets, GSE22220 (Illumina humanRef-8 v1.0 expression / human v1 miRNA Bead chip) and GSE19783 (Agilent 014,850 whole genome / 019,118 miRNA microarray) were used^12,13^. On February 16, 2018, files containing mRNA and miRNA data from primary breast cancer tumor samples were downloaded. GSE22220 mRNA and miRNA data, and GSE19783 mRNA data in log2 form were normalized. GSE19783 miRNA data were normalized using the quantile normalization method and Limma package in R. After normalization, samples were matched by mRNA and miRNA sample IDs. Quantile was used as a threshold to determine AR status. AR expression greater than quartile 1 or 25th percentile was considered AR-positive. AR expression lower than quartile 1 or 25th percentile was considered AR-negative. Log2 fold change was calculated for miRNA expression.

### Molecular subtype classification

A clinical file was obtained for the TCGA breast cancer dataset. Immunohistochemical (IHC) status for ER, PR, and Her2 were considered for subtype classification, and AR mRNA status was used as described above. The samples were classified as luminal A (ER- and/or PR-positive and HER2 negative), luminal B (ER- and or PR-positive and HER2-positive), HER2 enriched or HER2 type (ER-negative, PR-negative, and HER2-positive), TNBC (ER-negative, PR-negative, and HER2-negative), AR-positive TNBC (ER-negative, PR-negative, HER2-negative, and AR-positive), QNBC (ER-negative, PR-negative, HER2-negative, and AR-negative)^14–17^.

### Data analysis

Heatmaps were generated with R by use of clinical parameters for clustering. P-adjusted and *p*-values were calculated for differential gene expression analysis using the DESEQ2 package in R. Venny 2.1 was used for visualization in the form of Venn diagrams (https://bioinfogp.cnb.csic.es/tools/venny/index.html). JMP statistical analysis software was used to prepare box plots and for statistical analysis for the non-parametric Wilcoxon test. In some cases, a two-tailed t-test was used to obtain statistical significance between groups. Pathway Studio MammalPlus was used to assess miRNA interactions with genes or proteins using a network builder tool (https://www.pathwaystudio.com/). A graph pad was also used for data visualization. The KM plotter was used to download the data prepared for miRNAs for AAs and CAs in the overall breast cancer TCGA dataset using a pan-cancer tab^18^. The data for Kaplan Meier plots for miRNAs for molecular subtype TNBC patients in TCGA or METABRIC datasets were downloaded and using the miRNA breast cancer tab^19^. The data was downloaded from for each plot separately. and then using JMP software (Cary, NC) KM curves were plotted for each miRNA analysis). KM plots for breast cancer mRNAs were used for the miRNA target genes for survival analysis by the TNBC Pietenpol subtypes^20,21^. The probability of survival was calculated using the KM method, and log-rank tests were used to calculate the *p*-values.

## Results

### miRNA profiles in AR-positive versus AR-negative tumors

Using DESEQ2 analysis of TCGA to reveal miRNA signatures of AR-negative versus AR-positive cancers, we identified 45 miRNAs that were differentially expressed and clustered by AR status and subtype (Fig. S2A and Table S4). Among these 45 miRNAs, AR-negative cancers demonstrated upregulation of mir-519a-1, mir-519a-2, and mir-516a-1, which are associated with the miRNA-19 cluster, and mir-17, mir-18a, mir-17, and mir-20a, which are associated with the miRNA17-92 cluster. In contrast, mir-449a/b and mir-135a-1/2 were downregulated in AR-negative cancers. The miRNA signatures of AR-negative cancers were similarly expressed in QNBC, and miRNA expression within AR-positive cases was associated with luminal A/B and TNBC AR-positive cases (Fig. S2A). When compared to miRNA differential expression for AR negative versus AR positive cases in TCGA and GEO datasets (GSE19783 and GSE22220), 15 miRNAs overlapped among the three independent datasets (Fig. S2A).

**Figure 1:**
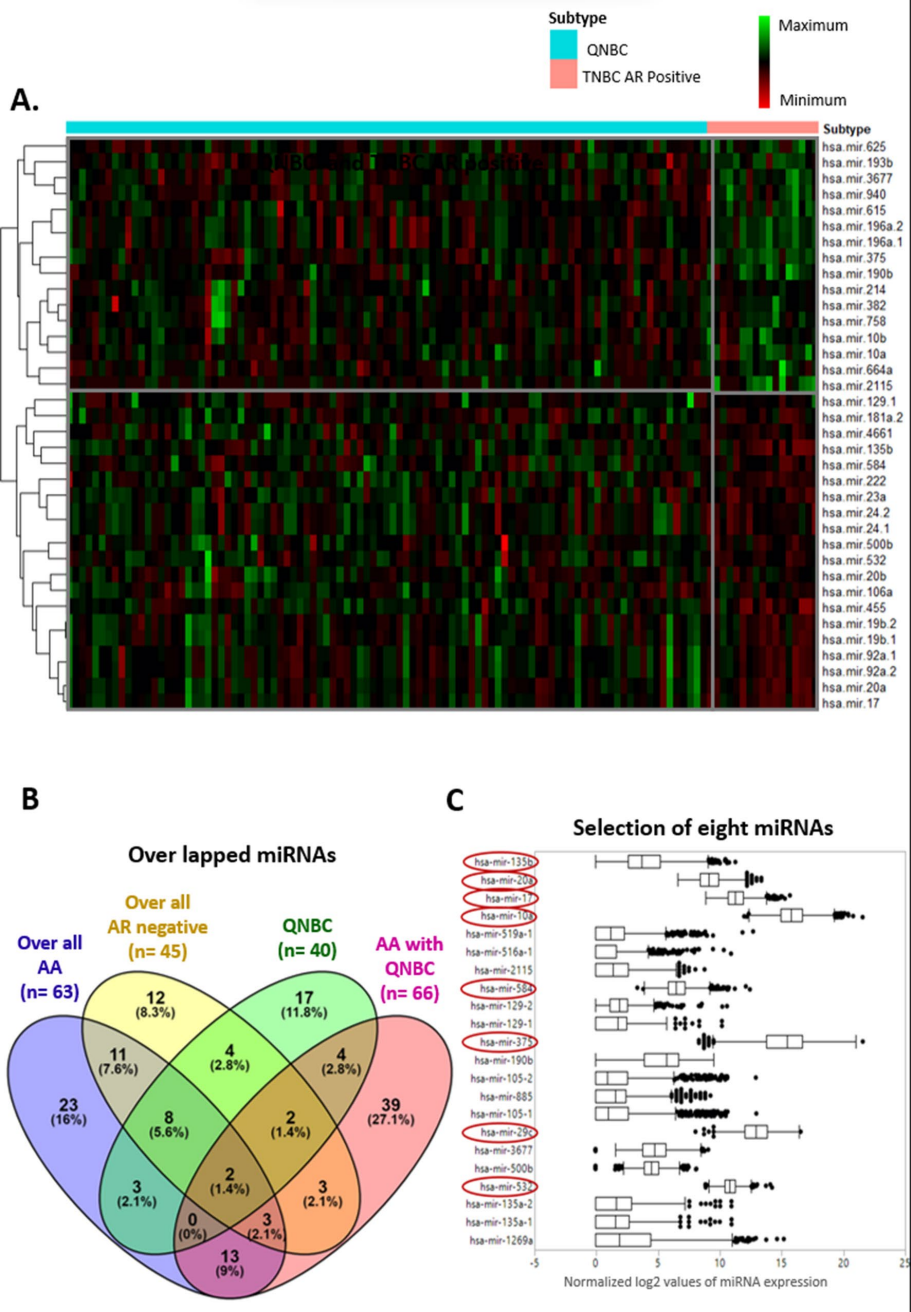
The miRNA signature of QNBCs differs from that of TNBCs that are AR-positive, and QNBC tumors of AAs exhibit a distinctive miRNA signature. A list of 40 miRNAs dysregulated in QNBCs and eight highly expressed miRNAs were selected for further analysis. (**A**) miRNAs differentially regulated in QNBCs (n = 97) compared to AR-positive TNBCs (n = 17). The heatmap shows the expression of 40 miRNAs that are dysregulated in QNBCs. The fold change of these miRNAs is shown in Table S5. The columns of the heatmap represent the samples, and the rows represent the miRNAs. The expression of miRNA is in the log2 form of the normalized miRNA expression. The heatmap is clustered by subtype. miRNAs in the upper cluster are downregulated in QNBCs, and lower clusters of miRNAs are higher in QNBCs. Presented is a list of 16 miRNAs that are downregulated in QNBCs, and a list of 20 miRNAs that are upregulated in QNBCs. These miRNAs may be associated with a basal-like phenotype. (**B**) A butterfly plot showing the overlapped miRNAs in between four differential miRNA expressions. The list of miRNAs differentially expressed in four DESEQ2 analyses was obtained. Presented is a list of 45 miRNAs dysregulated in AR-negative samples compared to AR-positive overall samples and a list of 40 miRNAs dysregulated in QNBCs compared to AR-positive TNBCs. Presented are a list of 63 miRNAs dysregulated in AA breast cancers compared to breast cancers of CAs, a list of 66 miRNAs dysregulated in AA QNBCs, and a list of 22 selected miRNAs overlapping by race and QNBC. (**C**) A forest plot showing the log2 values of miRNAs as represented in the X-axis. The Y-axis presents the 22 selected miRNAs. Eight miRNAs whose expressions are high were selected for further analysis. Red color shapes highlight those eight miRNAs.

To determine miRNAs that are associated with race and QNBCs, we performed four DESEQ2 analyses (Fig. 1B and S1A). First, we found there are 45 miRNAs differentially expressed in overall AR-negative versus -positive cases, which accounted for 24% and 76% of down- and upregulated miRNAs, respectively (Table S4 and Fig. S2A). Second, 63 miRNAs were differentially expressed in overall AA versus CA cases (Table S6 and Fig. S3), which accounted for 54% and 46% down- and upregulated miRNAs, respectively. Third, 40 miRNAs differentially expressed in QNBCs as compared to AR-positive TNBCs showed 37% and 63% down- and upregulation, respectively (Table S5 and Fig. 1A). Lastly, in QNBCs, 66 miRNAs were differentially expressed in AAs versus CAs (Table S7 and Fig. S4) with proportional up- and downregulation of 85% and 15%, respectively. Moreover, we evaluated the overlap between the differential miRNA expression for results from four DESEQ analyses (Table S4, S5, S6, and S7) by Venn diagram (Fig. 1B).

To determine miRNAs differentially expressed in QNBCs, we performed DESEQ2 analysis for QNBC versus TNBC AR-positive tissues, for which 40 miRNAs were found to be dysregulated in QNBCs (Fig. 1A). h mir-17 and 20a demonstrated elevated expression in overall AR-negative cases, AA cases, and QNBC cases. A majority of the downregulated miRNAs in AA QNBCs are located on chromosome 14 (Fig. S5 and Table S7). Additionally, 13 miRNAs appear to be race specific as they are differentially expressed in both overall AR negative and QNBCs groups irrespective of AR status (Fig. 1B).

**Figure 2:**
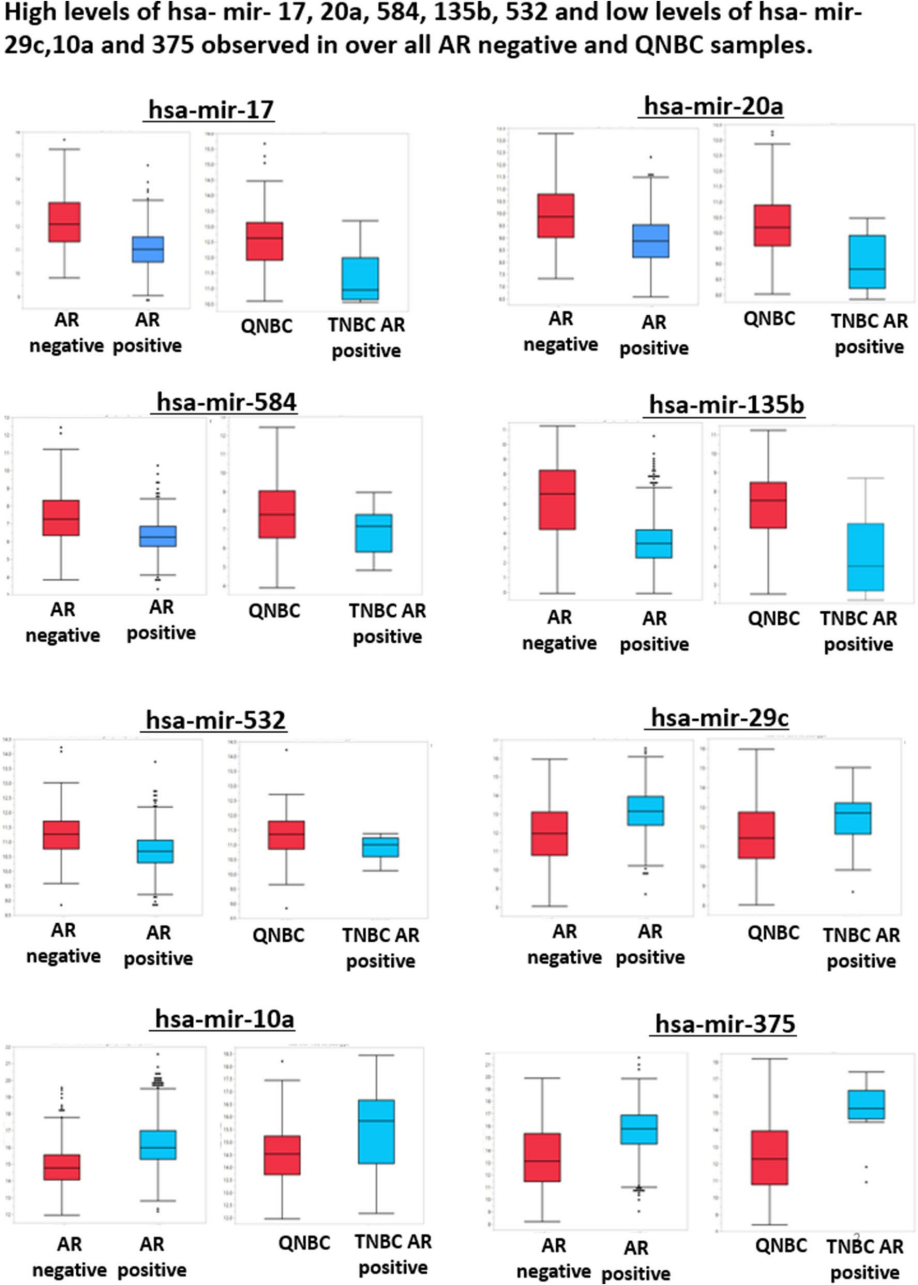
High levels of mir-17, mir-20a, mir-584, mir-135b, and mir-532 and low levels of mir-29c, mir-10a, and mir-375 are present in overall AR-negative breast cancers and QNBCs. Eight highly expressed miRNAs were selected; the box plot shows the expression of these miRNAs in AR-negative (n = 267) and AR-positive (n = 823) primary breast cancers (nonparametric Wilcoxon test *p* < 0.05). For AR-negative versus AR-positive overall the X-axis represents the AR status (negative or positive), and the Y-axis represents the miRNA expression normalized and log2 scale for the corresponding miRNA. For QNBC versus TNBC AR-positive, the box plot shows the expression of these miRNAs in QNBC primary breast cancers (n = 97) and AR-positive TNBC (n = 17) samples (non-parametric Wilcoxon test P < 0.05). The X-axis represents the subtype (QNBC or TNBC AR-positive), and the Y-axis represents the miRNA expression normalized and the log2 scale for the corresponding miRNA. There were high levels of mir-17, mir-20a, mir-584, mir-135b, and mir-532 and low levels of mir-29c, mir-10a, and mir-375 in overall AR-negative samples and QNBC.

Out of 22 miRNAs, we selected eight (8) highly expressed miRNAs for further analysis (Fig. 1C). To evaluate the expression pattern of eight highly expressed miRNAs by AR status, race in overall samples, and QNBCs, we evaluated their expression by box plots, which showed high levels of hsa-mir-17, 20a, 584, 135b, and 532, and low levels of hsa-mir-29c,10a, and 375 in overall AR-negative samples (Table S1 and Fig. 2). Further, there were high levels of hsa-mir-17, 20a, 584, 135b, and 532 and low levels of hsa-mir-29c,10a, and 375 in QNBCs relative to AR-positive TNBCs (Table S2 and Fig. 2).

**Figure 3:**
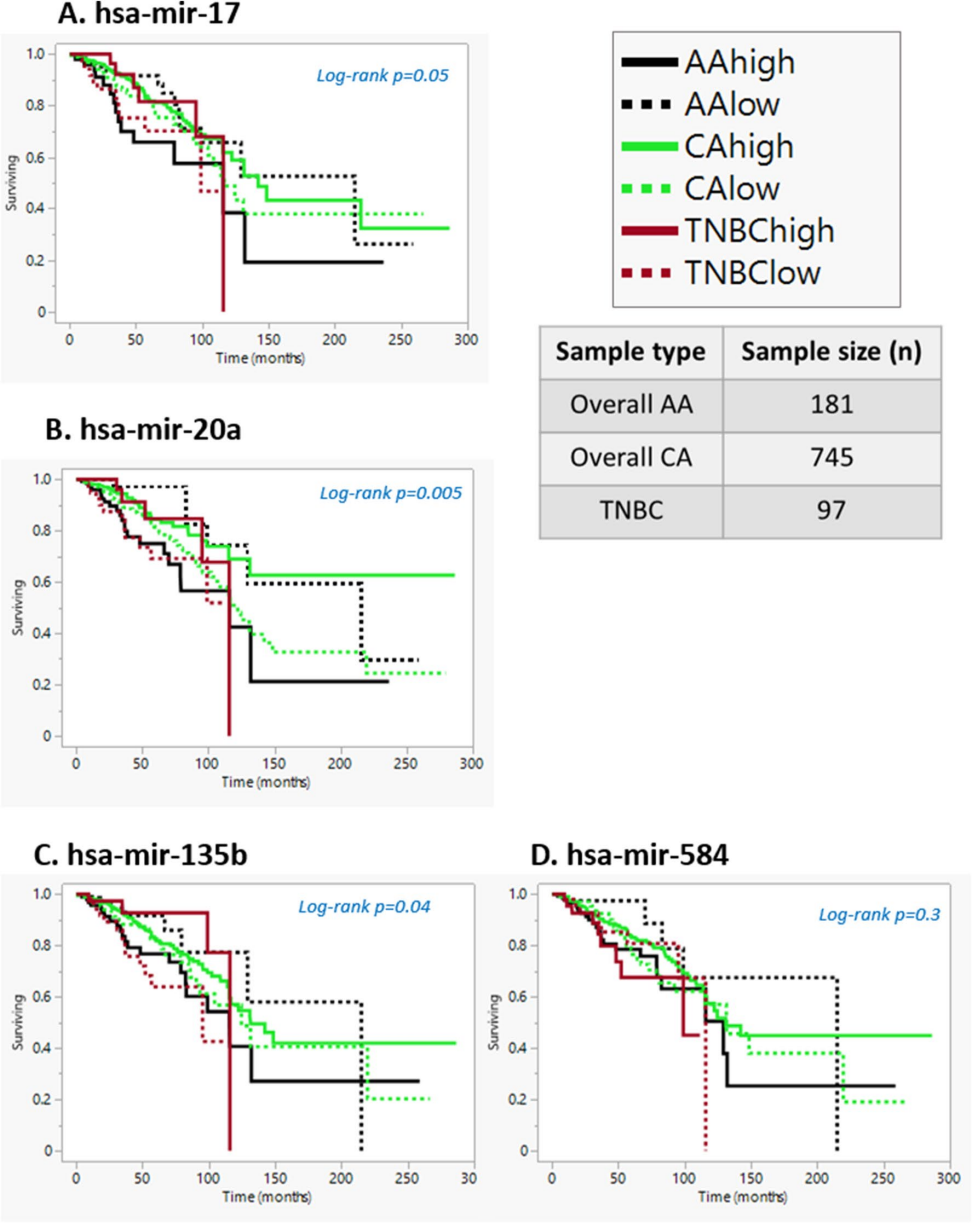
Survival analysis for mir-17, mir-20a, mir-135b, and mir-584 in overall AAs (n = 181), Overall CA (n = 745), and TNBCs (n = 97). AA patients with high levels of hsa-mir-17 or mir 20a or mir135b have lower survival, however, there is a trend that CA and TNBC patients with low levels of has-mir-17 or mir 20a or mir135b have lower survival rates. AA and TNBC patients with high levels of hsa-mir-584 seem to have lower survival, however, there is a trend that CA patients with low levels of has-mir-584 have lower survival rates.

### miRNAs that have a differential impact on the survival of AA patients

**Figure 4:**
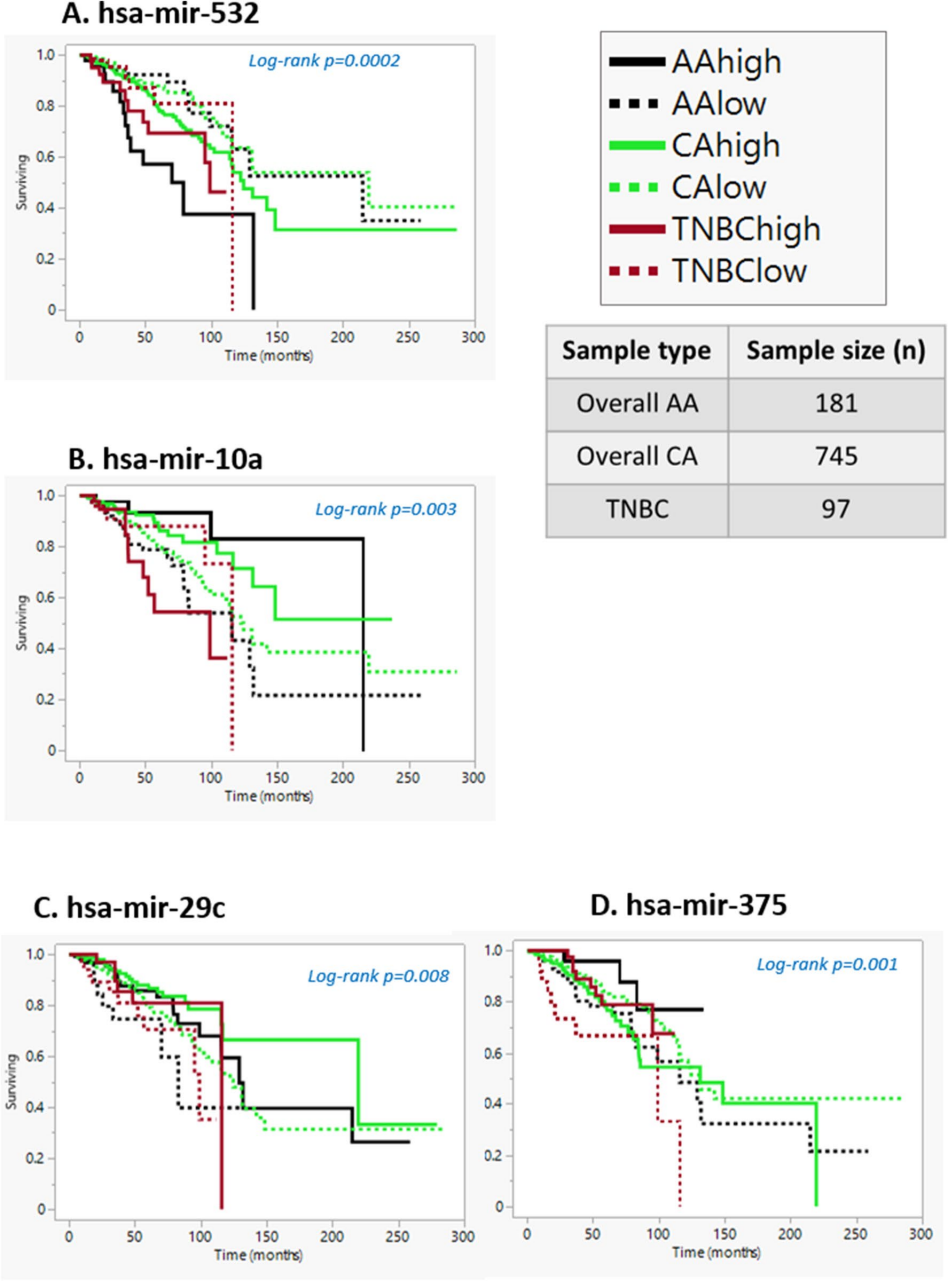
Survival analysis of mir-532, mir-10a, mir-29c, and mir-375 for overall AAs (n = 181), Overall CA (n = 745), and TNBCs (n = 97). Irrespective of race or subtype hsa-mir-532 high levels seem to have lower survival rates AA, CA and TNBC patients. AA and CA patients with low levels of hsa-mir-10lower survival, however there is a trend that TNBC patients with high levels of has-mir-10a seems to have lower survival rates. Irrespective of race or subtype hsa-mir-375 and hsa-mir-29c low levels seems to have lower survival rates AA, CA and TNBC patients.

To determine the correlation of miRNAs (hsa-mir-17, hsa-mir-20a, hsa-mir-135b, hsa-mir-584, hsa-mir-532, hsa-mir-10a, hsa-mir-375, and hsa-mir-29c) with overall survival for AA and CA patients with tumors across all breast cancer subtypes, we utilized data from the Breast cancer TCGA dataset (Figs. 3A–D and 4A–D). AA breast patients that had high levels of mir-17, 20a, 135b, and 584subtype had lower survival rates compared to CA patients. Further, all patients, irrespective of race, with high levels of mir-532 (Fig. 4A) and low levels of mir-10a (Fig. 4B) and miR-29c (Fig. 4C) had lower survival rates. However, AA cancers, regardless of subtype, and TNBCs with low levels of mir-375 appeared to be associated with lower survival, compared to high levels of mir-375 in CA cancers that appeared to be associated with a lower survival rate (Fig. 4D). Thus mir-375 may have an opposite function in AA and CA.

To validate our findings in an independent dataset, we performed survival analysis in TNBC patients from the METABRIC dataset, which contains a higher number of TNBC patients than in the TCGA. We observed higher expression of mir-17, mir-20a, mir-135b, mir-532 and mir-375 and lower expression of mir-584, mir-10a and mir-29c seems to be associated with lower survival (Figs. S9 and S10).

### Genes associated with miRNAs are dysregulated in QNBCs

**Figure 5:**
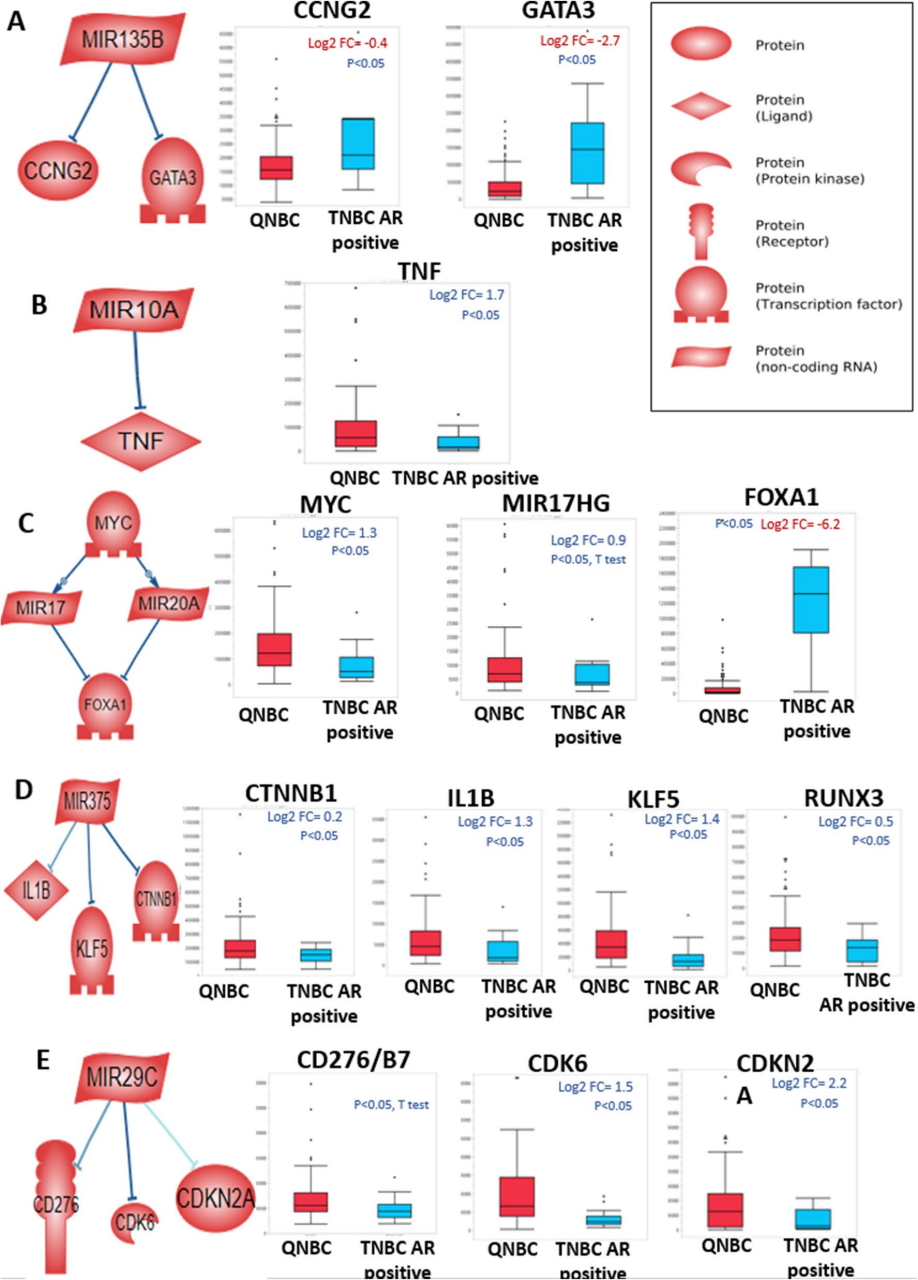
Genes associated with miRNAs are dysregulated in QNBCs. Predicted targets of miRNAs were obtained by use of Pathway Studio. The expression of gene targets was evaluated in QNBCs, and the correlations of miRNAs and genes were obtained for overall TCGA samples**.** Non-parametric Wilcoxon test *p*-values are presented. (**A**) mir-135b targets CCNG2 and GATA3, the expressions of which were lower in QNBCs. (**B**) mir-10a targets TNFα, the expression of which was higher in QNBCs. (**C**) Pathway Studio networks revealed that MYC positively regulates mir-17 and -20a, which target FOXA1. MYC and mir-17HG were higher in QNBCs, and FOXA1 was lower in QNBCs. (**D**) Pathway Studio analysis showed that mir-375 targets IL-1B, KLF5, and CTNNB1. Literature reports show that mir-375 targets RUNX family proteins^55^. We observed that IL1B, KLF5, CTNNBA, and RUNX3 are upregulated in QNBCs. (**E**) From network analysis, Pathway Studio revealed that mir-29C targets CD276, CDK6, and CDKN2A. In QNBCs, CD276 (*p* < 0.05 by T-test and *p* = 0.05 by Wilcoxon), CDK6, and CDKN2A are upregulated.

Since miRNAs control gene expression, we next sought to determine the genes associated with the expression of the selected miRNAs in QNBCs while making a statistical comparison with AR-positive TNBCs. To assess gene targets, we used the Pathway Studio database to find a mRNA gene that relates to each differentially expressed miRNA through network enrichment analysis. Pathway Studio analysis revealed that the mir-135b targets CCNG2 and GATA3, and both demonstrated lower mRNA expression in QNBC compared to AR-positive TNBC (Fig. 5A). Further analysis of CPTAC proteomic data confirmed that CCNG2 and GATA3 protein levels is negatively correlate with mir-135b expression as well. (Table S3). mir-10a, which targets TNFα has a higher mRNA expression level in QNBCs as compared to AR-positive TNBCs (Fig. 5B), and this was further confirmed at the protein level d (Table S3).

MYC, a transcription factor, appears to regulate the expression of MIR17HG in addition to mir-17 and mir-20a. Expression of MYC and MIR17HG are higher, and FOXA1 expression was lower in QNBC relative to AR-positive TNBC patients . Furthermore, mir-17 and 20a positively correlate with MYC and MIR17HG, and, conversely, negatively correlated with FOXA1 in overall primary tumor samples (Fig. 5C and Table S3). miR-375, which targets CTNNB1, IL1B, KLF5, and RUNX3, was higher in QNBCs as compared to AR-positive TNBCs in mRNA level (Fig. 5D). Lastly, mir-29c, which targets CDK6, CDKN2A, B7H3, and CDKN2A, was higher in QNBCs as compared to AR-positive TNBCs. Lastly mir-29c negatively correlated with CD276/B7H3, CDK6, and CDKN2A at both the RNA and proteomic expression levels (Fig. 5E and Table S3).

Expressions of gene targets were evaluated in all BC samples by all subtypes (Fig. S6A) and in TNBC samples by comparing TNBC AR positive Versus QNBC subtype (Fig. S6B) by heatmap. The miRNA-targets/ gene expression showed a pattern of lower expression of HOXB3, HIF1AN, CCNG2, FOXA1, and GATA3 in QNBC, nd higher expression was observed for CD276/B7H3, CTNB1, TGFBR2, IL1B, TNF, KLF5, CDK6, RUNX3, MYC, MIR17HG, CDKN2A and E2F3 in the QNBC subtype (Fig. S6).

To further understand the biological function of the miRNAs dysregulated in QNBC, pathway analysis was performed. The miR system database^22^ was utilized for the eight selected miRNAs dysregulated in Overall AA, AR negative and QNBC (hsa-miR-20a, hsa-miR-135b, hsa-miR-584, hsa-miR-17, hsa-miR-532, hsa-miR-10a, hsa-miR-29c, and hsa-miR-375) for pathway interactome database. Interestingly, the top 6 hits from the miRNA pathway analysis appear to target proteins that have an important role in the p53 effector pathway. The p53 pathway is known to orchestrate many cellular pathways including DNA damage response mechanisms (Table S10). Additionally, Pathway analysis was done for list of miRNAs for (1) AR positive versus AR negative (Table 4), (2) QNBC versus TNBC AR positive (Table S5), (3) AA versus CA overall (Table S6) and (4) AA versus CA QNBC (Table S7). The nuclear SMAD 2/3 and P53 signaling pathway is one of the top hits (Table S11, S12, S13 and S14). Previous studies have shown that SMAD 3 forms a complex with BRCA1 during DNA damage and TGF beta/SMAD3 counteracts BRCA1 dependent repair of the DNA damage^23^. Therefore, the DNA damage pathway seems to be effected in QNBC.

### Association of AR, mir-29c, and CD276/B7H3 in QNBCs

**Figure 6:**
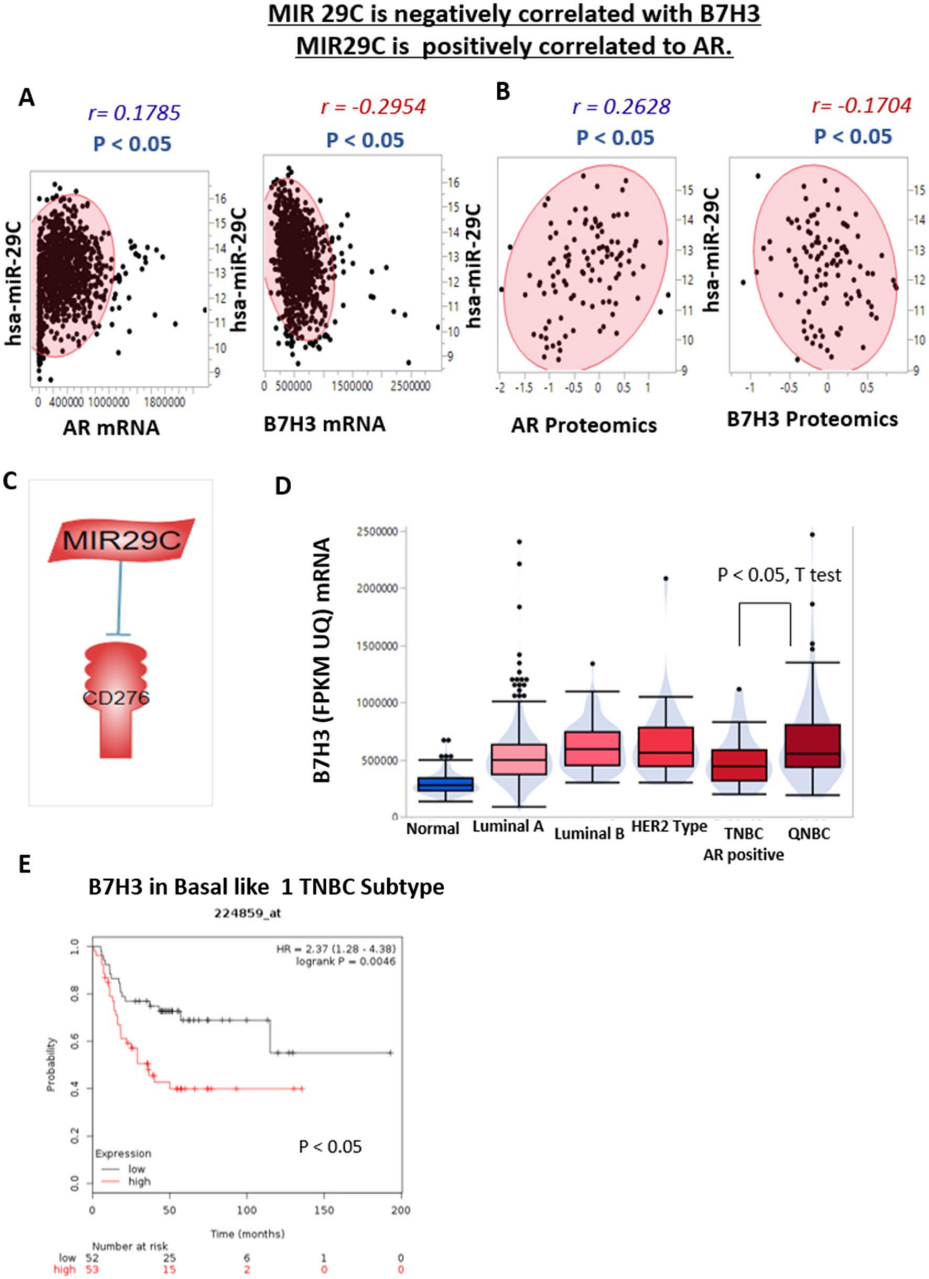
Correlations of AR, miR29C, and B7H3. AR positively correlated with mir-29C, which negatively correlates with B7H3. B7H3 is high in QNBCs compared to AR-positive TNBCs. In TNBCs, high B7H3 is associated with lower survival of patients with the basal-like 1 subtype. Non-parametric t-test *p*-values are indicated. (**A**) Correlation scatter plots show the Pearson correlation coefficients. In mRNA data (n = 1083) for TCGA overall primary tumor samples, mir-29C positively correlates with AR and negatively correlates with B7H3. (**B**) In proteomics data for TCGA overall primary tumor samples (n = 105), mir-29C positively correlates with AR and negatively correlates with B7H3. (**C**) Pathway Studio analysis revealed that mir-29C targets CD276/B7H3, which is located on the cell membrane and known to be clinically relevant. (**D**) The box plot shows the mRNA gene expression of B7H3 in FPKM UQ units. As compared to healthy tissues, B7H3 expression was higher in primary breast cancer tissues of different BC subtypes. B7H3 expression was higher in QNBCs as compared to AR-positive TNBCs. (**E**) Survival analysis was accomplished with KM Plotter with breast cancer datasets for B7H3 (probe ID 224859_at) by the Pietenol subtypes of TNBCs. Patients with high B7H3 in basal-like 1 TNBCs have lower survival.

Since mir-29c had a significant correlation with survival probability for patients with TNBCs, we next determined if, in QNBC patients, the loss of AR regulates mir-29c expression as well as its gene target CD276/B7H3. CD276/B7H3, which is located on the cell membrane, is clinically relevant due to its overexpression in tumor tissues and limited expression in normal tissues, coupled with an immunoregulatory role in tumor microenvironment modeling and expansion^24^. In TCGA mRNA data for overall primary tumor samples (n = 1083), mir-29c positively correlated with AR and negatively correlated with CD276/B7H3. Similarly, in TCGA whole primary tumor samples proteomics data (n = 105), mir-29c positively correlated with AR and negatively correlated with CD276/B7H3 (Fig. 6), indicating that AR may upregulate mir-29c and subsequently downregulate its target CD276/B7H3. Plus, AR mRNA is positively correlated to AR proteomics level, when compared in same BC sample (n = 108) (Fig. S8).

As compared to apparently healthy tissue, CD276/B7H3 expression was higher in primary tumor tissues in various cancer subtypes. CD276/B7H3 expression was higher in QNBC patients as compared to AR-positive TNBC patients (Fig. 6). In overall samples, CD276/B7H3 mRNA expression was higher in breast cancers of AA patients compared to CA patients. Also, there was a similar pattern in CPTAC proteomic data. AA patients had higher CD276/B7H3 as compared to CA patients (*p* = 0.08), but the difference was not statistically significant (Fig. S7). Lastly, we performed survival analyses using breast cancer datasets for the CD276/B7H3 (probe ID 224859_at) subtype of TNBC. Patients with high CD276/B7H3 in basal-like 1 TNBC had lower overall survival (Fig. 6E). Further, high CD276/B7H3 was associated with lower survival for patients with the BL-2, mesenchymal, and mesenchymal stem-like subtypes as well (Fig. S7^1^). There was a similar pattern for both AA and CA patients with high CD276/B7H3, although the difference was not statistically significant (Fig. S7).

## Discussion

The versatility of miRNAs at physiological and pathological levels makes them indispensable factors for many types of cancer, including mammary cancers. This is corroborated by the fact that they have a function in racial cancer disparities based on diagnosis, prognosis, disease progression, and response to various therapeutic modalities^25^. For instance, mir-655 expression positively correlates with COX-2 in genetically diverse breast cancer cells grown as spheroids, thereby linking it with stem-like cells^26^. Clinically, miRNAs such as miR-155, miR-222, miR-125b, and miR-21 are associated with tumor resistance to the most common systemic treatments, qualifying them as potential predictors of response to breast cancer therapeutics^27^. Our group recently demonstrated^28^ that miRNAs like miR-17-5p, miR-432, miR-663a, and miR-1225 are implicated in the TNBC-to-QNBC transition, further underscoring their roles in cancer disparities. miRNA expression levels impact the levels of downstream genes or clinically relevant biomarkers. However, in previous literature, it is unknown how miRNA signatures differ among AA with CA patients relative to AR-positive TNBCs and QNBC subtypes. Consequently, the present communication helps to fill this knowledge gap by establishing relevant baseline information.

Around 70% of TNBCs are QNBCs^1,29,30^. AR appears to add prognostic benefits to determine which tumors are aggressive and which are non-aggressive. In our previous report, we demonstrated that QNBCs of AA women have distinctive basal and immune gene signatures^1^. In a follow-up study using IHC of clinically relevant protein markers, we found that metastatic QNBCs had higher levels of EGFR expression and lower levels of PTEN and the Wnt signaling inactivator, TLE3^1,14^. These studies comparing miRNA signatures and mRNA-mediated genomic expression uncovered differences in QNBCs and AR-positive TNBCs that could explain the aggressive character of this disease.

To understand the regulatory networks associated with QNBCs, we assessed miRNA expression. We found high levels of has-mir-17, mir-20a, mir-584, mir-135b, and mir-532 and low levels of mir-29c, mir-10a, and mir-375 in overall AR-negative, overall AA, and QNBC cases. Only a few studies have evaluated miRNA in association with AR status in breast cancers. In 2017, Shi et al.^31^compared AR-negative and AR-positive cell lines; the results did not coincide with our findings for patients. Breast cancer cell lines treated with dihydrotestosterone showed an increase in the expression of mir-363 and mir-21^17,32^. Although these studies were limited to cell lines, they provide evidence of AR-regulated miRNAs. In our results, however, mir-21 and mir-363 did not change with AR status. Our findings are consistent with those of Gong et al*.*, who reported that breast cancers of AA patients have lower levels of mir-10a^33^. Moreover, Sugita et al. compared miRNA expression patterns in a separate TNBC cohort of AAs (n = 27) and non-Hispanic whites (n = 30). Consistent with our results, hsa-mir-17-5p and hsa-mir-18a-5p were upregulated in AA TNBCs; however, in contrast, mir-532-3p was low in AA TNBCs^34^. Telonis et al*.*^35^found multiple miRNA isoforms and showed that tRNA-derived fragments were associated with TNBCs and disparities. Relative to these results, our results seem to be different because we focused on investigating miRNA expression in QNBCs and on disparities.

Based upon our findings and the literature, miRNAs are altered in QNBCs, resulting in a basal-like phenotype, and causing alterations in the immune response. The target genes of miRNAs and transcription factors responsible for miRNA transcription showed an association with the basal-like phenotype and immune function^36^. In the present study, we identified an association of the miRNA/gene network that leads to aggressive disease in QNBCs, as highlighted below.

Firstly*,* mir-135b was elevated in QNBCs, overall AR-negative breast cancers, and AA breast cancers. The target genes of miR-135b, involved in the inhibition of the EMT (CCNG2) and inflammatory responses (GATA3) are lower in QNBCs^37,38^. Also, Uva et al*.*^39^categorized mir-135b as upregulated in the basal-like subtype compared to the non-basal-like subtype. Secondly*,* mir-17 and 20a expressions were higher in overall AR-negative and QNBC samples. MYC positively regulates mir-17 and 20a^40^,^41^. MYC expression was higher in QNBCs, and FOXA1 was lower in QNBCs. Loss in FOXA1 expression is associated with the basal subtype^42,43^. Also, MYC and FOXA1 are among the PAM50 genes, which we have previously been found in QNBC tumors^1^. This evidence indicates that AR loss in QNBCs could cause an increase in MYC that further increases transcription of mir-20a and -17 and down-regulation of FOXA1. In consequence, loss of FOXA1 and an increase in MYC could lead to a basal-like phenotype in QNBC patients. Thirdly*,* mir-375 is downregulated in overall AR-negative breast cancers and QNBC patients. A decrease in mir-375 expression is associated with the basal subtype and malignant breast cancer^44^. We observed that mir-29c expression was low in all l AR-negative cases . CD276/B7H3 is a clinically relevant target of mir-29c^45^. Patients with high CD276/B7H3 in basal-like cancers have a lower survival rate^46^. Finally*,* our findings demonstrate that mir-10a is similarly downregulated in AA patients that are AR-negative. mir-10a is a regulator of the nuclear factor kappa-light-chain-enhancer of activated B cells (NF-κB) signaling pathway^47,48^. There is a negative association between mir-10a and TNFα^49,50^. TNFα expression was higher in QNBC patients, and TNFα negatively correlated with mir-10a, suggesting that, in QNBC patients, a loss in mir-10a could cause an increase in TNFα.

**Figure 7:**
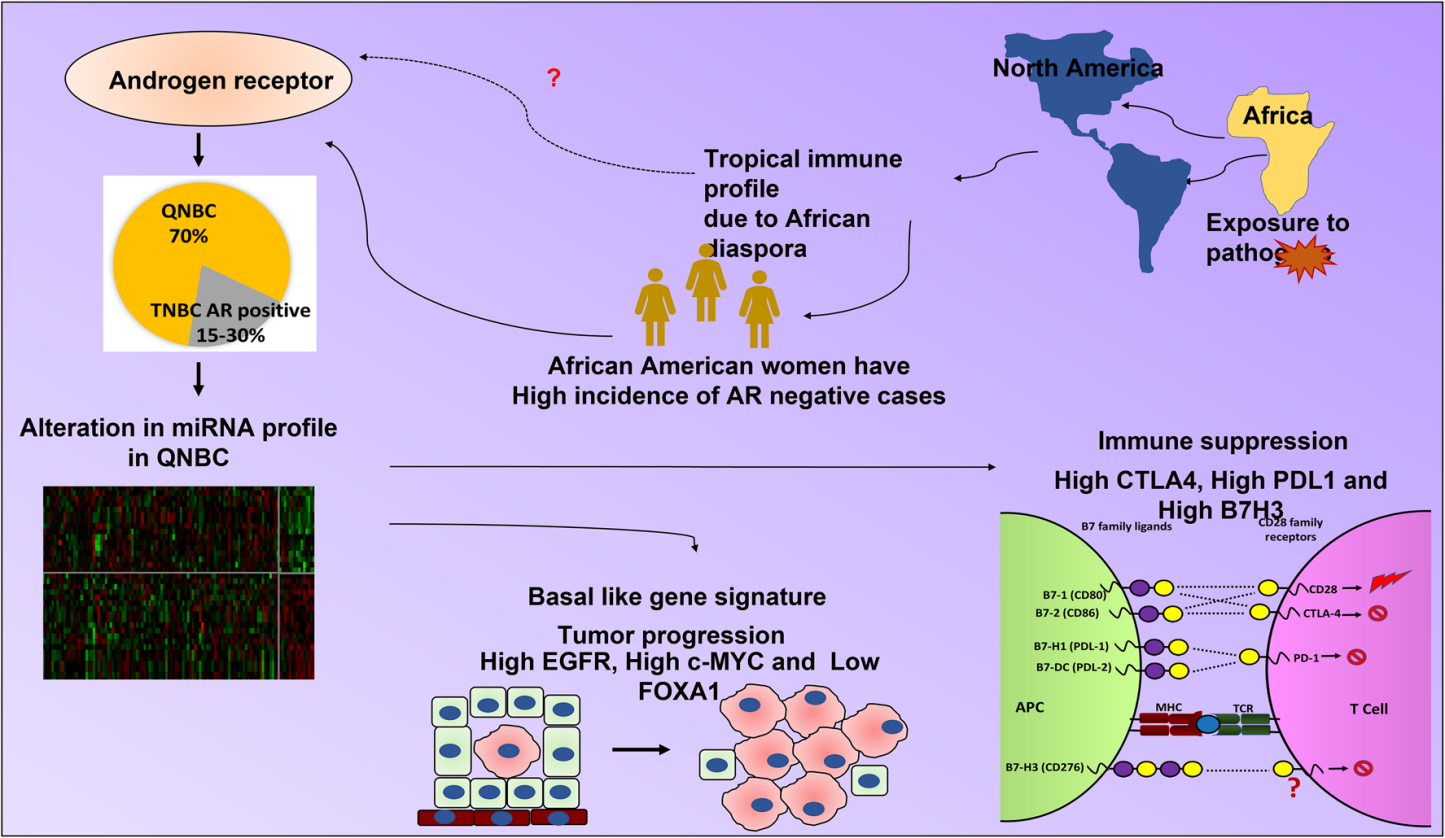
Summary figure/graphical abstract indicating that altered miRNA and gene signatures in QNBCs and cancers of AA patients could be the basis for the immune suppression and tumor progression of QNBCs.

Geographical location and ancestry may contribute to immune response. African ancestry seems to be associated with aggressive type breast cancer^51–53^. African American women seem to have a higher incidence of AR-negative cases (Fig. 7). miRNA and mRNA profiles suggest that QNBC patients have basal-like gene signatures and immune suppression. In a nutshell, our findings suggest that AR may play role in genetic ancestry and could play a role in the regulation of gene expression in aggressive type breast cancer. Our study has several strengths, including the large sample size, validation in independent datasets by AR IHC in TNBC samples, a comprehensive analysis of eight highly expressed miRNAs, evaluation of miRNA targets, and assessment of survival probability. Our results further provide the underlying molecular mechanisms of the basal-like gene signature in QNBCs and breast cancers of AA women (Fig. 7). Therefore, to strengthen our findings, research on copy number variations of the miRNA host genes, AR binding patterns near the transcription start sites of the miRNAs, methylation, and possible single nucleotide polymorphisms of the miRNAs are needed. It’s worth noting that loss of AR in TNBC patients has been reported to be associated with loss of DNA repair gene RAD51AP1^54^. Although this study focused on miRNA–mRNA immune related genes, we did observe enrichment of both SMAD2/3 signaling and p53 related pathway genes in AA QNBC tumors would suggest a role for DNA Damage Repair (DDR) genes. Thus, there should be future investigations into the ancestry-related correlation of miRNAs and miRNA host genes associated with loss of AR expression, particularly in African American QNBC patients. Nevertheless, our results provide evidence that miRNAs have a function in breast cancer and highlight the importance of using miRNAs as biomarkers for clinical risk assessment and as potential therapeutic targets.

A limitation of this study is the complexity of patient-derived data. Survival probability depends on external factors such as the time of disease progression and vital status. The patients undergo different treatments, and the procedures may have an impact on vital status or gene expression. So, there is a discrepancy in the TNBC cohort's survival probability in TCGA and METABRIC datasets. For miRNAs mir-17, mir-20a, mir-135b, mir-584, mir-10a, and mir-375, there is a discrepancy between datasets for TNBC cases. For both datasets, however, mir-532 and -29c were consistent. We speculate that this could be due to the inclusion of several patients with AR-like TNBCs, or to different breast cancer stages associated with patients in the TNBC group. Also, the directionality of miRNAs (3p or 5p) is not included in TCGA data. Lastly, we cannot rule out other factors that can influence the vital status and miRNA signatures, such as patient characteristics or breast cancer stages, and exposure to environmental factors.

## Conclusion

In our previous study, IHC of metastatic tumors showed that the percentage of tumors positive for EGFR was higher, and those positive for PTEN and TLE3 were lower in QNBCs compared to TNBCs. This evidence showed that QNBC is a consistent subtype and that AR is a reliable marker to predict metastasis and recurrence. Furthermore, we established that QNBC tumors tend to be basal-like, with elevated EGFR expression and higher rates of Wnt signaling activity. We also evaluated miRNA expression in breast cancers of AA women with the QNBC subtype. By use of TCGA data, we found that the expression of miRNAs in QNBCs differs from AR-positive TNBC cases. High levels of mir-17, mir-20a, mir-584, mir-135b, and mir-532 and low levels of mir-29c, mir-10a, and mir-375 were present in overall AR-negative breast cancers and QNBCs. In addition, in QNBCs, miRNA-associated genes such as MYC are upregulated, and FOXA1 is downregulated. We confirmed that miRNAs and their associated gene signatures are altered in QNBCs and breast cancers of AA patients, which could explain the distinctive basal-like and immune gene signatures in QNBCs of AAs. In summary, our study demonstrated that AR negativity is associated with breast cancers of AA women and that QNBC is an aggressive subtype with an altered gene signature.

## Supplementary Information

Supplementary Information 1.

Supplementary Information 2.

## Data Availability

The data previously published in TCGA (https://portal.gdc.cancer.gov/), and GEO were used for this study. We did not use any new data or raw data generated by us. The miRNA, mRNA, and proteomics data for breast cancer from TCGA (https://portal.gdc.cancer.gov/) were used. Additionally, the microarray data used is available in GEO (https://www.ncbi.nlm.nih.gov/geo/query/acc.cgi?acc=GSE22220) for GSE22220 (Illumina humanRef-8 v1.0 expression / human v1 miRNA Bead chip) and GSE19783 (https://www.ncbi.nlm.nih.gov/geo/query/acc.cgi). These datasets were cited appropriately in the “Materials and methods” section of this manuscript.
