# Supplementary material for: MicroRNAs within the Basal-like signature of Quadruple Negative Breast Cancer impact overall survival in African Americans
Source: Sci Rep. 2022 Dec 22;12:22178. doi: 10.1038/s41598-022-26000-9 (PMC9780260; doi:10.1038/s41598-022-26000-9)
Supplement: Supplementary file 1 — Supplementary Information 1. [file 41598_2022_26000_MOESM1_ESM.docx]

**MicroRNAs within the basal-like signature of Quadruple Negative Breast Cancer Impact Overall Survival in African Americans.**

Anusha Angajala ^1,4^; Hughley Raymond^1^; Aliyu Muhammad^1,7^; Md Shakir Uddin Ahmed^1,8^; Saadia Haleema^4^; Monira Haque^4^; Honghe Wang^1^, Moray Campbell^12^, Rachel Martini^2^; Balasubramanian Karanam^1^; Andrea G. Kahn ^6^; Deepa Bedi^1^, Melissa Davis^2^; Ming Tan ^5^; Windy Dean-Colomb^1,3^; Clayton Yates^1.,9.10, 11*^.

^1^Department of Biology and Center for Cancer Research, Tuskegee University, Tuskegee, AL 36088.

^2^Department of Surgery, Weill Cornell Medicine, New York, NY 10021.

^3^Department of Hematology/Oncology, Piedmont Hospital, Newnan, GA 30265.

^4^Department of Pathology, University of South Alabama, Mobile, AL 36604.

^5^Graduate Institute of Biomedical Sciences and Research Center for Cancer Biology, China Medical University, Taichung, Taiwan 406040

^6^Department of Pathology, The University of Alabama at Birmingham, Birmingham, AL 35249-7331.

^7^Department of Biochemistry, Faculty of Life Sciences, Ahmadu Bello University, Zaria, Kaduna State, Nigeria, 810107.

^8^Bangladesh Council of Scientific and Industrial Research (BCSIR), Dhaka, Bangladesh

^9^Department of Pathology, Johns Hopkins School of Medicine, Baltimore, MD 21218, USA

^10^Sidney Kimmel Comprehensive Cancer Center, Johns Hopkins University School of Medicine, Baltimore, MD 21218, USA

^11^Department of Urology, Johns Hopkins University School of Medicine, Baltimore, MD 21218, USA

^12^Pharmaceutics and Pharmaceutical Chemistry, College of Pharmacy, The Ohio State University, Columbus, OH 43210

**Running Title:** Implications of miRNAs in quadruple negative breast cancer and racial disparities.

**Keywords:** QNBC (Quadruple negative breast cancer), AR (Androgen receptor), AA (African American), CA (Caucasian), BC (Breast cancer).

**Corresponding Author:**

Clayton Yates,

John R. Lewis Professor of Pathology

Professor of Pathology, Oncology, Urologic-Oncology

Director for Translational Health Disparities and Global Health Equity Research

Program Co-Leader for Cancer Genetics and Epigenetics

Sidney Kimmel Comprehensive Cancer Center

Johns Hopkins School of Medicine

The Bunting-Blaustein Cancer Research Building 1
1650 Orleans Street - Room 1M44

Baltimore, MD 21287-0013

Contact: cyates10@jhmi.edu


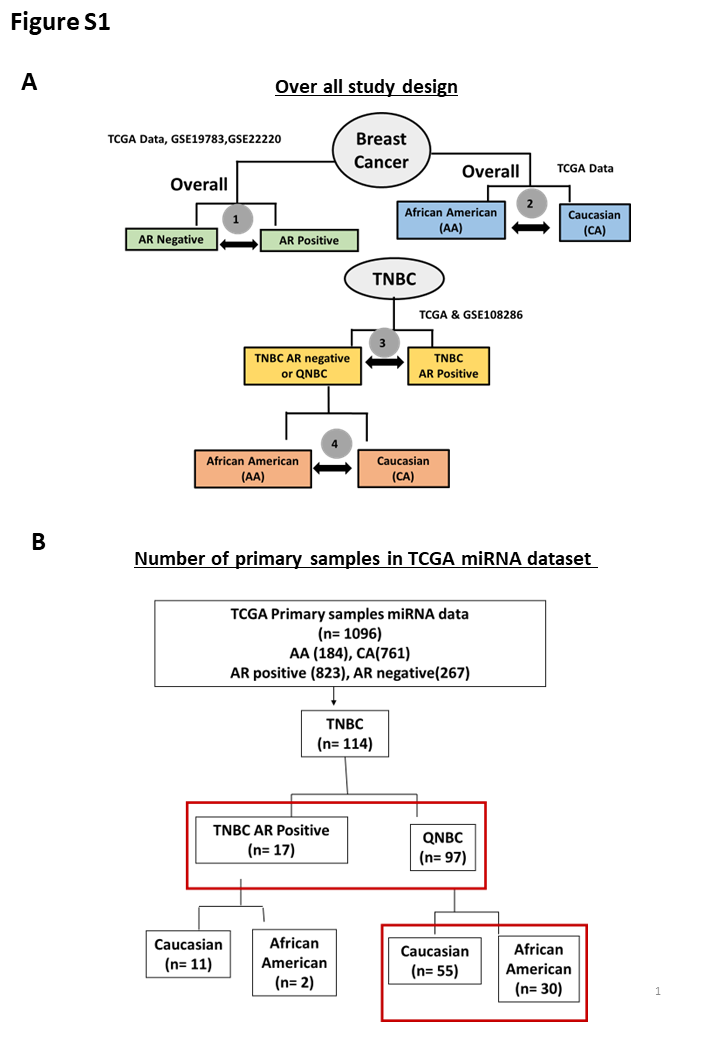


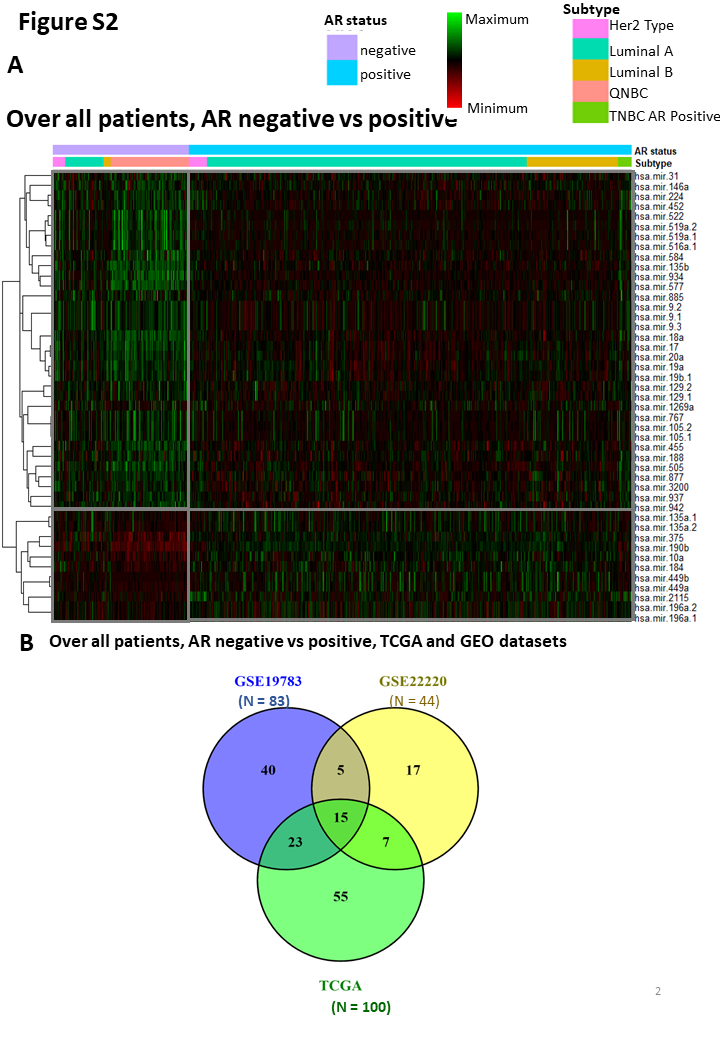


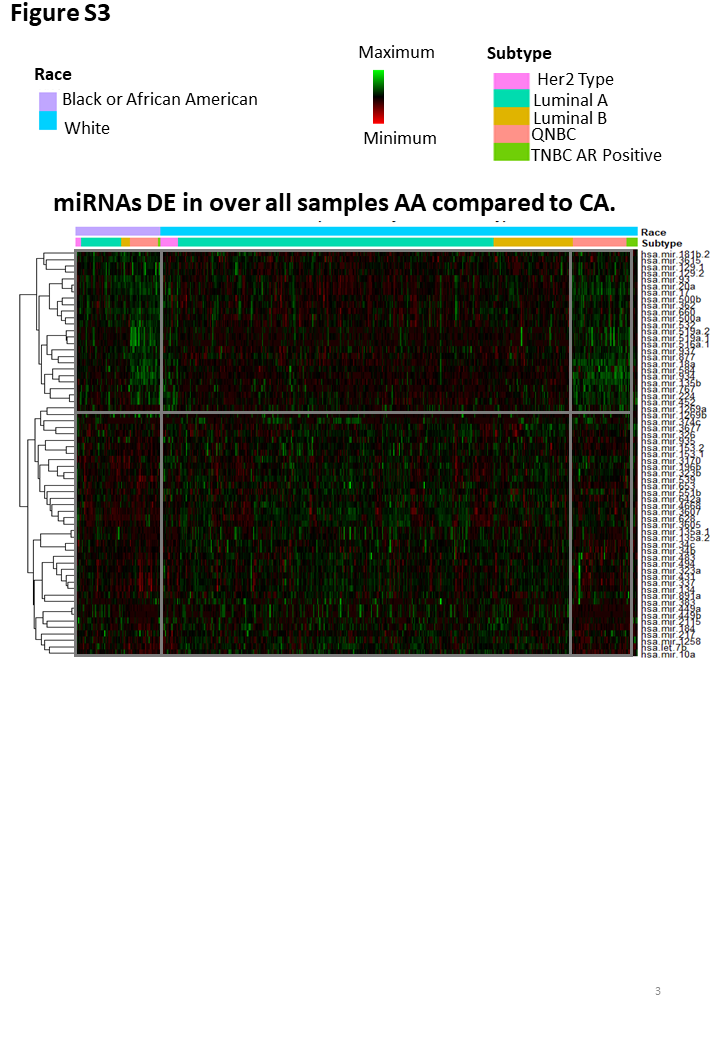


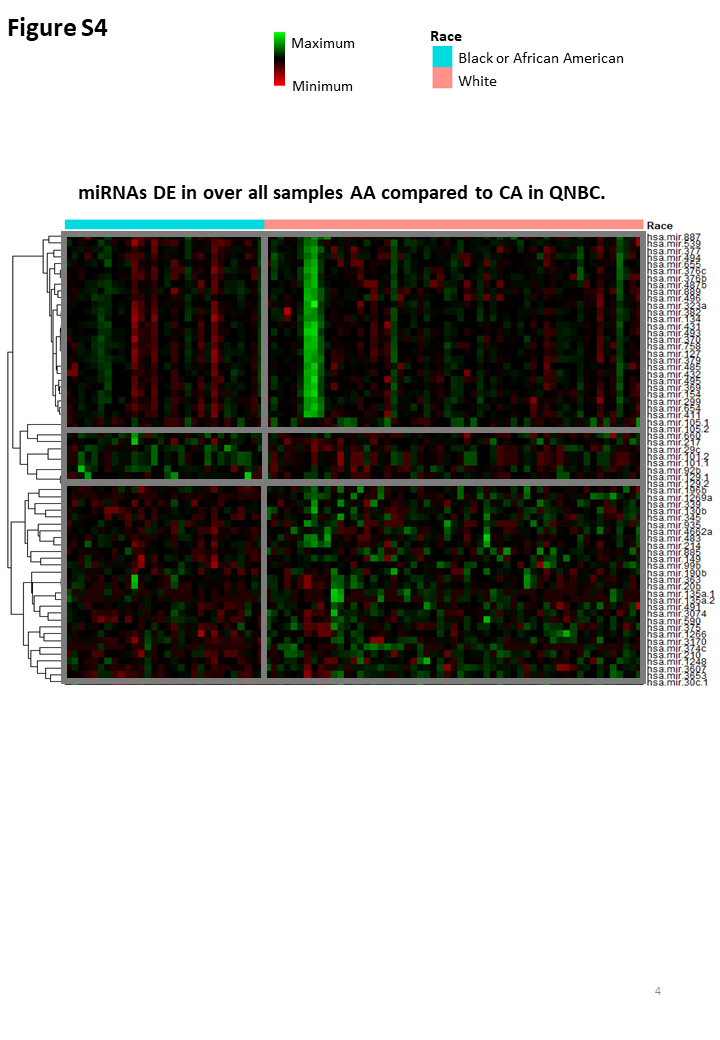


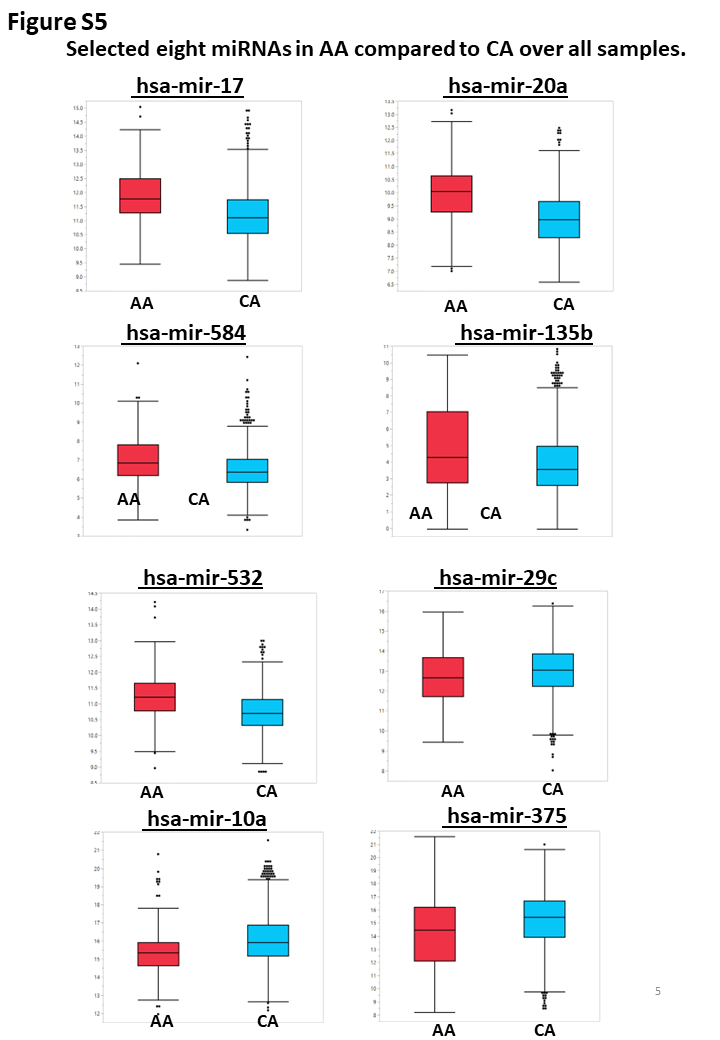


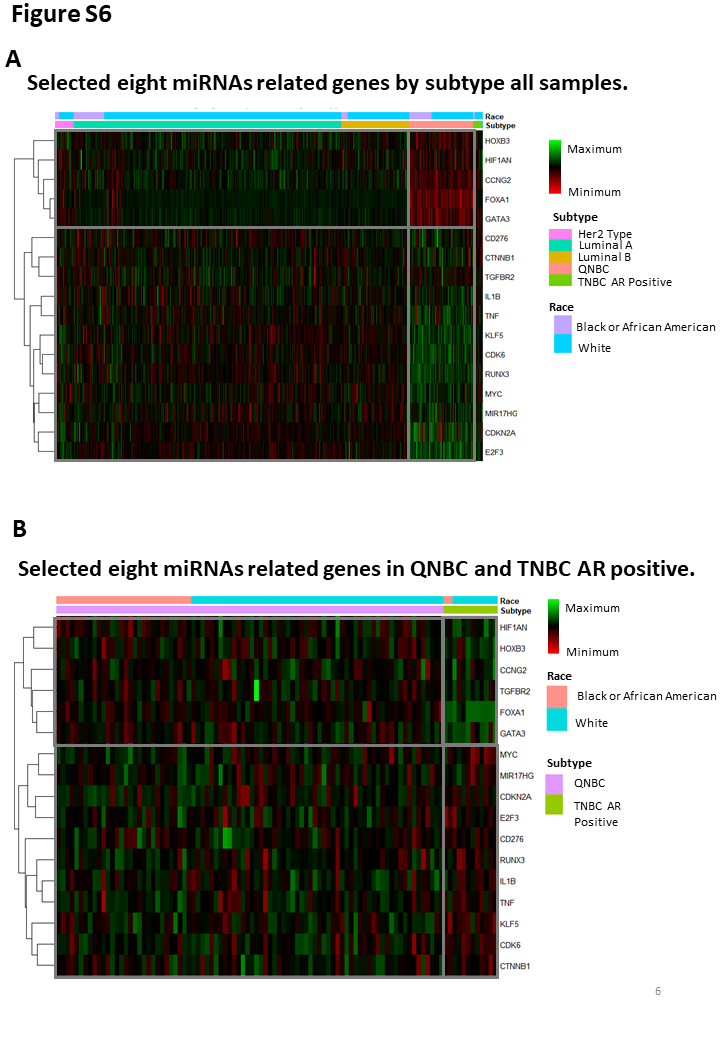


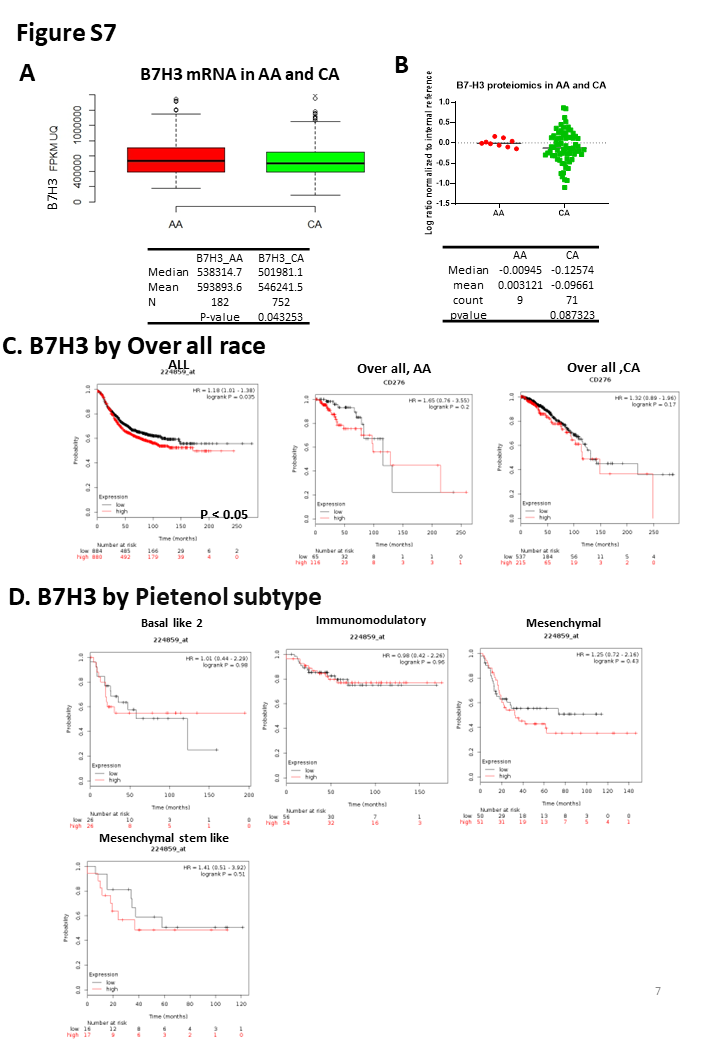


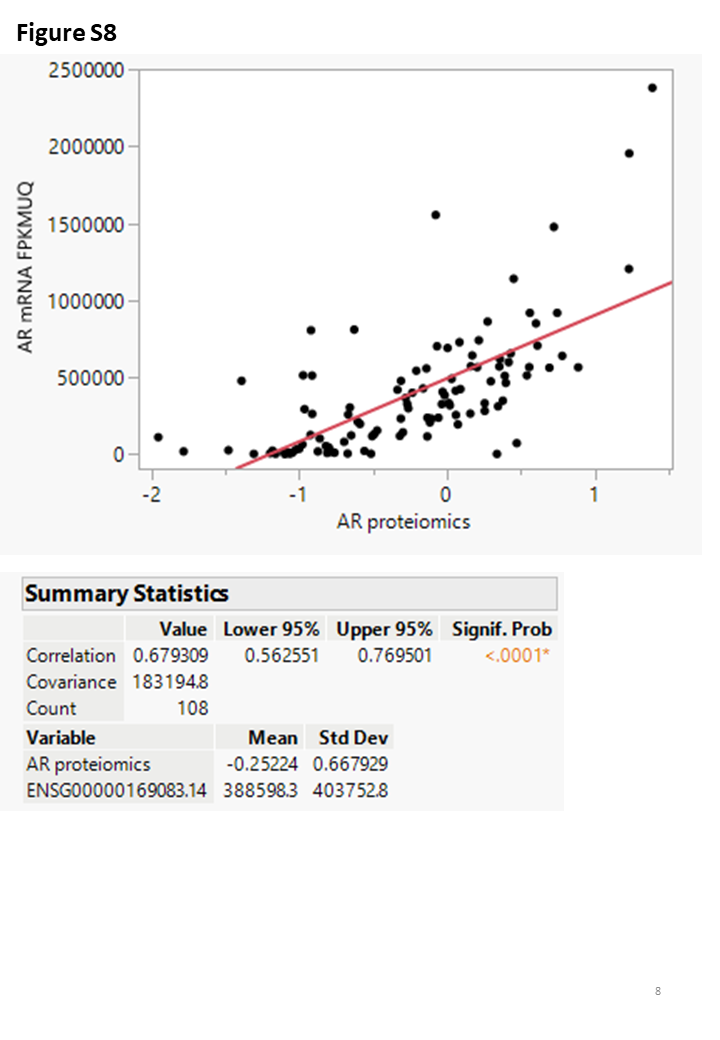


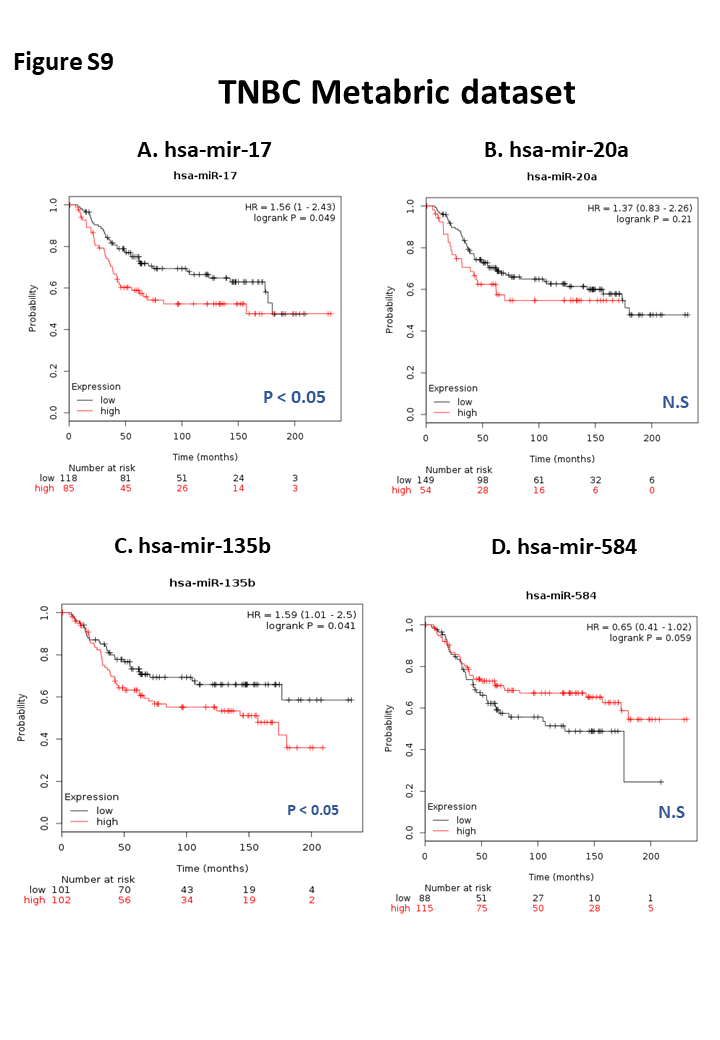


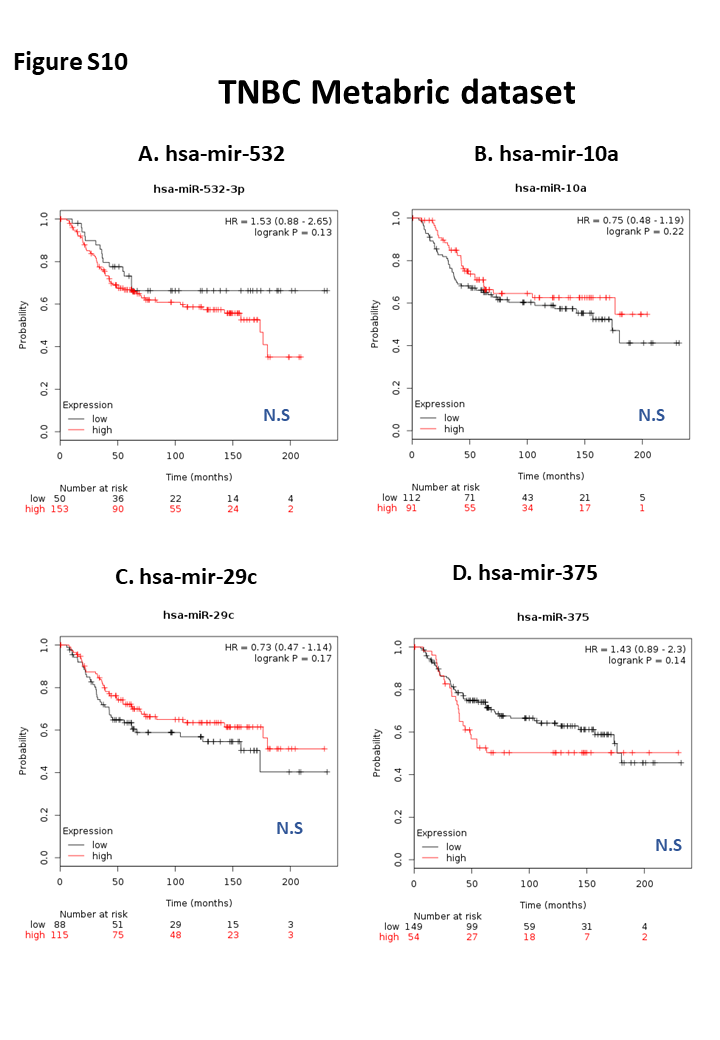


**SUPPLEMENTAL FIGURE LEGENDS**

**The heatmaps represent miRNA expression in overall patients (AR negative vs positive, AA vs CA) and miRNA expression differences in QNBC AA vs QNBC CA. Also, miRNA predicted target gene expression was evaluated.** The flow chart represents the overall study design and numbers of samples in the TCGA miRNA primary sample dataset (Figure S1). Most AR-negative cases have miRNA expression like QNBCs and the Her2 type; AR-positive cases have miRNA expression like luminal A/B and TNBC AR-positive types (Figure S2). List of 63 miRNAs differentially regulated in AA breast cancers compared to CA breast cancers (Figure S3). List of 66 miRNAs differentially regulated in QNBCs of AAs relative to QNBCs of CAs (Figure S4). High levels of mir-17, mir-20a, mir-584, mir-135b, and mir-532 and low levels of mir-29c, mir-10a and mir-375 are present in breast cancers of AAs (Figure S5). The predicted targets of miRNAs were obtained by Pathway Studio. Expressions of gene targets were evaluated in QNBCs and overall TCGA samples (Figure S6). B7H3 expression is high in AA breast cancers compared to CA breast cancers (Figure S7). To understand the correlation of the AR gene in mRNA level to the protein level, the correlation was done for TCGA RNA seq data mRNA AR gene in (FPKM UQ) to proteomics data for TCGA for AR in the same patient sample. From this analysis, we found that AR mRNA is positively correlated to AR proteomics data. (Correlation =0.67) (Figure S8). Survival analysis for the eight miRNAs was performed in KM Plotter for Breast cancer metabric dataset for TNBC subtype (Figure S9 and S10).

**SUPPLEMENTAL TABLE LEGENDS**

**The tables represent the list of miRNAs differentially expressed in overall AR negative vs positive, QNBC vs TNBC AR-positive, overall AA vs CA, and QNBC AA vs QNBC CA. Also, the tables represent the LOG2 fold change for the eight selected miRNAs.** High levels of mir-17, mir-20a, mir-584, mir-135b, and mir-532 and low levels of mir-29c, mir-10a, and mir-375 are present in overall AR-negative cases (Table S1). High levels of mir-17, mir-20a, mir-584, mir-135b, and mir-532 and low levels of mir-29c, mir-10a, and mir-375 are present in QNBCs (Table S2). miRNA to related gene correlation coefficient (Table S3). List of 45 miRNAs differentially expressed in AR-negative cases as compared to AR-positive cases in all samples (Table S4). A list of 40 miRNAs dysregulated in QNBCs compared to AR-positive TNBCs (Table S5). A list of 63 miRNAs dysregulated in breast cancers of overall AAs compared to CAs (Table S6). A list of 66 miRNAs dysregulated in AA QNBCs compared to CA QNBCs (Table S7). High levels of mir-17, mir-20a, mir-584, mir-135b, miR-532, and low levels of mir-29c, mir-10a, and mir-375 are present in AA breast cancers (Table S8). Demographic information (Breast cancer characteristics categorized by AR status and race) for TCGA miRNA dataset used in this study can be found in Table S9. To see pathway effected by these eight miRNAs (hsa-miR-20a, hsa-miR-135b, hsa-miR-584, hsa-miR-17, hsa-miR-532, hsa-miR-10a, hsa-miR-29c, and hsa-miR-375), the miRsystem database was used for the eight selected miRNAs for pathway interactome database. Please refer to Supplementary table S10 for the pathway rankings table. Additionally, Pathway analysis was done for list of miRNAs for 1) AR positive vs AR negative (table S4), 2) QNBC vs TNBC AR positive (Table S5), 3) AA vs CA overall (Table S6) and 4) AA vs CA QNBC (Table S7). The nuclear SMAD 2/3 and P53 signaling pathway is one of the top hits (Table S11, S12, S13 and S14).
